# Posterior Uterine Rupture Causing Fetal Expulsion into the Abdominal Cavity: A Rare Case of Neonatal Survival

**DOI:** 10.1155/2011/426127

**Published:** 2011-10-20

**Authors:** K. Navaratnam, P. Ulaganathan, M. A. Akhtar, S. D. Sharma, M. G. Davies

**Affiliations:** Southport and Ormskirk Hospital, NHS Trust, Town Lane, Kew, Southport, Merseyside PR8 6PN, UK

## Abstract

*Introduction*. Uterine rupture is a potentially catastrophic complication of vaginal birth after caesarean section. We describe the sixth case of posterior uterine rupture, with intact lower segment scar, and the first neonatal survival after expulsion into the abdominal cavity with posterior rupture. *Case Presentation*. A multiparous woman underwent prostaglandin induction of labour for postmaturity, after one previous caesarean section. Emergency caesarean section for bradycardia revealed a complete posterior uterine rupture, with fetal and placental expulsion. Upon delivery, the baby required inflation breaths only. The patient required a subtotal hysterectomy but returned home on day 5 postnatally with her healthy baby. *Discussion*. Vaginal birth after caesarean section constitutes a trial of labour, and the obstetrician must be reactive to labour events. Posterior uterine rupture is extremely rare and may occur without conventional signs. Good maternal and fetal outcome is possible with a prompt, coordinated team response.

## 1. Introduction

Uterine rupture is a recognised complication of vaginal birth after caesarean section (VBAC), with serious maternal and fetal outcomes. It is conventionally attributed to the structural compromise of the uterine scar, as depicted by the term “trial of scar.” The incidence of uterine rupture during VBAC is the lowest after one previous caesarean section, with spontaneous, progressive labour not requiring augmentation. Prostaglandins and oxytocics to induce and augment labour increase the risk of uterine rupture 2-3-fold. We present a case of atypical uterine rupture of the posterior uterine wall, with induced, augmented labour, and a discussion of the case characteristics and the existing literature.

## 2. Case Presentation

A 38-year-old gravida 3, para 2 requested VBAC. She previously had a spontaneous vaginal delivery at term, followed by unstable lie in her second pregnancy, necessitating elective lower segment caesarean section at 37 weeks. There were no existing medical or antenatal problems, and she had uneventful consultant-led care throughout her third pregnancy. She attended counselling regarding mode of delivery, with explicit discussion of postmaturity management, and specific risks of uterine rupture during spontaneous labour, induction, and augmentation. She chose to proceed with a trial of labour, with induction of labour if indicated. Third-trimester ultrasound demonstrated a posterior placenta, and well-grown fetus, with cephalic presentation.

At 42 weeks of gestation she was admitted, for Prostaglandin induction, according to protocol. On admission, her Bishop's score was 4, and 1 mg prostaglandin E2 was administered vaginally. After 12 hours she was 2 cm dilated, and partially effaced, transferred to delivery suite for continuous fetal cardiotocography (CTG) and artificial rupture of membranes (ARM). After ARM, uterine activity remained mild and incoordinate and induction of labour was continued with oxytocin. She established in labour, with oxytocin titrated to uterine activity, with regular moderate contractions and a 3-minute pain-free interval. After 6 hours of Oxytocin her cervix was effaced and 5 cm dilated, the fetal head was in the left occipitotransverse position, and station was −2. 18 minutes later she had a brisk antepartum haemorrhage (APH) of 150 mL, associated with persistent fetal bradycardia to 80 bpm. She was rapidly assessed by the specialist registrar. Uterine scar rupture was suspected, and a grade one emergency caesarean section conducted, under general anaesthetic, with the consultant obstetrician in attendance. 

Upon peritoneal entry, there was a large haemoperitoneum and the uterus was well contracted with an intact lower segment scar. The fetus and placenta had been expelled via a posterolateral uterine wall rupture, both were free in the posterior peritoneal cavity. A live male infant was promptly delivered and handed to the neonatal team; the decision to delivery interval was 13 minutes.

Initially the infant made no respiratory effort but maintained a heart rate of greater than 100 bpm. Three cycles of five inflation breaths were required. Apgar scores were 2 at 1 minute, 6 at 5 minutes, and 10 at 10 minutes. Venous and arterial umbilical cord pHs were 6.988 with Base excess −13.4 and 6.958 with Base Excess −10.1, respectively. The birthweight was 3.26 kg.

The 8 cm posterior uterine rupture extended into the left lateral uterine wall, and the left infundibulopelvic ligament was completely avulsed from its attachment. The infundibulopelvic vessels had retracted into the retroperitoneal space and were bleeding persistently. Due to severe uterine trauma with loss of pelvic attachments and haemorrhage, a subtotal hysterectomy was required. A senior consultant was called to assist, and the consultant anaesthetist and haematologist were alerted. After completing the subtotal hysterectomy, the retroperitoneal space was opened, to mobilise the infundibulopelvic vessels. The estimated intraoperative haemorrhage was 2000 mL. The patient had high-dependency obstetric care for 24 hours postoperatively, haemoglobin intraoperatively was 5.0 g/dL and a transfusion of 4 units of packed red cells was commenced. The events were carefully explained by the operating consultant, and the patient recovered remarkably quickly. She was discharged home with her baby on day five postnatally and comprehensively debriefed by the obstetric team six weeks postnatally.

## 3. Discussion

Uterine rupture is a defect involving the full thickness of the myometrium and uterine serosa or myometrial disruption extending to the bladder or broad ligament [1]. Uterine dehiscence describes a partial thickness defect, with intact uterine serosa, which may progress to rupture [1]. Uterine rupture occurring in an unscarred uterus is very rare, with an incidence of 0.5–2.0/10000, largely confined to labouring multiparas [2]. However, isolated occurrences of prelabour and primiparous uterine rupture are described [3–5]. 

During vaginal birth after caesarean section (VBAC) the Royal College of Obstetricians and Gynaecologists quotes an incidence of uterine rupture of 22–74/10000, which should be referred to when counselling women regarding mode of delivery [6, 7]. Induction and augmentation of labour after caesarean section is associated with a 2-3-fold increased risk of uterine rupture, with an incidence of 140/10000 and 89/10000 for prostaglandin and syntocinon, respectively [7]. The incidence of hypoxic ischaemic encephalopathy and perinatal death associated with uterine rupture is 1/20 and 1-2/100, respectively [1]; maternal death occurs in 1/100000 cases [8].

In scarred uteri, the vast majority of uterine dehiscence and ruptures will occur via the uterine scar. The atrophic, inelastic nature of the scar renders it less adaptive to forces in labour, predisposing to scar rupture. However, a particularly rigid anterior lower segment may cause abnormal distribution of force. During retraction, the posterior wall may be excessively shortened and thinned due to the rigid anterior uterine scar, catalysing atypical uterine rupture via healthy tissue. Any factor compromising uterine structural integrity or causing abnormal distribution of force can precipitate uterine rupture. The site of uterine rupture is unpredictable and may be atypical. 

Five cases of posterior uterine rupture complicating vaginal birth after caesarean section have been published from 1997 to 2007 [9–13]. Recognised factors predisposing to uterine rupture during VBAC are induction or augmentation of labour, slow progress, labour dystocia, multiparity, fetal malposition, short interdelivery interval (less than 24 months), and placenta accreta [6]. In this case, the patient had one previous spontaneous vaginal delivery, followed by a caesarean section for unstable lie. Two other women were multiparous with parity of three or greater [9, 13], one with a previous twin pregnancy [13]. It is possible that uterine laxity or hyperdistension predisposes to atypical uterine rupture. 

In addition to our patient's case there are two reported cases of posterior uterine rupture with prostaglandin induction of labour, using prostaglandin E2 and dinoprostone [9, 11]. Three cases of posterior uterine rupture in spontaneous labour are also recorded [10, 12, 13]. In our patient's case the fetal head was occipitotransverse prior to rupture. Fetal malposition with an occipitoposterior position has previously contributed to posterior uterine ruptures, as has malpresentation with a transverse lie and dead fetus [12, 13]. Malposition alters the distribution of contractile force and increases labour dystocia; certain malpresentations cause uterine hyperdistension, which may also precipitate atypical uterine rupture.

In our experience, and that of two others, the first sign of posterior uterine rupture was a pathological CTG with persistent fetal bradycardia [10, 11]. CTG abnormalities are associated with 55–87% of uterine ruptures [14]. Other recognised signs of uterine rupture include loss of station of presenting part and new inefficient contractility [6]. 

APH, as in this case, often indicates uterine rupture and may occur in association with shoulder tip pain due to haemoperitoneum. APH was documented with one further case of posterior uterine rupture but was absent in 4/5 [13]. With posterior uterine rupture bleeding may be concealed, as described in two earlier cases where signs of hypovolaemia developed with each with a large concealed haemoperitoneum [9, 10]. Interestingly, in our patient's case maternal pulse and blood pressure remained within normal parameters despite massive uterine rupture, demonstrating the potentially misleading capacity for compensation in an otherwise fit patient. In 2/5 cases of posterior uterine rupture with VBAC women reported persistent abdominal pain [9, 13], in one case “breaking through” an effective epidural [13]. However, in our patient's case and three others there was no report of pain, despite no regional analgesia. 

This is the first case of fetal survival following fetal and placental expulsion from a posterior uterine rupture. Once delivered, the neonate required inflation breaths only, despite acidotic cord pHs. In this case the neonate was in excellent condition and did not require admission to the neonatal unit. There is one other documented case of neonatal survival, where the predelivery CTG was normal and the fetus was delivered from the uterine cavity, despite posterior rupture [13].

## 4. Comment

Induction and augmentation for VBAC have been integrated into daily obstetric practice. Uterine rupture remains a hazard and can develop rapidly, challenging the labour ward team. Despite known predisposing factors, uterine rupture is a rare event, and it is impossible to predict those women in whom it will actually occur. For this reason, VBAC should be considered a trial of labour. All women should be thoroughly risk-counselled antenatally, and the labour ward team should be vigilant particularly during induction and augmentation of labour. Obstetricians should adopt a flexible approach and be reactive to labour events. Posterior uterine rupture is extremely rare, conventional signs may be absent, and women may compensate well for massive concealed haemorrhage. However, good maternal and fetal outcomes are achievable, with a prompt coordinated team response and swift recourse to caesarean section.
